# Holding the PP2A and PP4 protein phosphatases at bay: emerging roles of FBXO42 and CCDC6

**DOI:** 10.1038/s44319-026-00849-7

**Published:** 2026-07-14

**Authors:** Jakob Nilsson

**Affiliations:** 1 Center for Epigenetic Cell Memory, Danish Cancer Institute, Copenhagen, Denmark; 2 Novo Nordisk Foundation Center for Protein Research, ICMM, University of Copenhagen, Copenhagen, Denmark

**Keywords:** Cancer, Post-translational Modifications & Proteolysis, Signal Transduction

## Abstract

Three new studies identify the ubiquitin ligase FBXO42 and the coiled coil protein CCDC6 as regulators of PP4 and PP2A.

**Figure:**
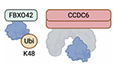

## Teaser

The Ser/Thr phosphoprotein phosphatases (PPPs, PP1-PP7) are major regulators of phospho-dependent signaling and precise regulation of their activity is essential for cellular homeostasis (Nilsson, 2019). Within the PPP family, PP2A, PP4 and PP6 holoenzymes share a common architecture composed of a catalytic subunit, a scaffolding subunit and distinct regulatory subunits conferring substrate specificity. A complex biogenesis pathway involving several proteins ensures the integrity of the catalytic subunit and proper formation of holoenzymes. In addition, regulated methylation of the terminal leucine residue on the catalytic subunit controls holoenzyme assembly.

Three new studies now identify the ubiquitin ligase FBXO42 and the coiled coil protein CCDC6 as regulators of PP2A and PP4, expanding the complexity of phosphatase regulation (Coassolo et al, 2026; Spangenberg et al, 2026; Yang et al, 2026). Although previous work had hinted at a connection (Barbosa et al, 2021; Jiang et al, 2022; Merolla et al, 2012; Ueki et al, 2019) the new studies provide direct evidence and mechanistic insight. FBXO42 is a poorly characterized member of the F-box only family, a large family of substrate adapter proteins for SCF ubiquitin ligases. FBXO42 is a nuclear protein and contains an N-terminal Skp1 binding domain and a C-terminal Kelch domain that has previously been shown to bind to the transcriptional regulator RBPJ (Jiang et al, 2022). In contrast, CCDC6 is a cytoplasmic protein that has been implicated in the DNA damage response through inhibition of PP4 (Merolla et al, 2012). Genome-wide CRISPR screening approaches conducted by DepMap reveal a strong correlation between FBXO42 and CCDC6 essentiality but the underlying molecular basis for this is unclear. Interestingly, ~15% of cancer cell lines are dependent on FBXO42 and CCDC6, and most of these cancer cell lines have defective p53 function. This dependency might be due to a mitotic vulnerability (Hoellerbauer et al, 2024).

**Figure 1:**
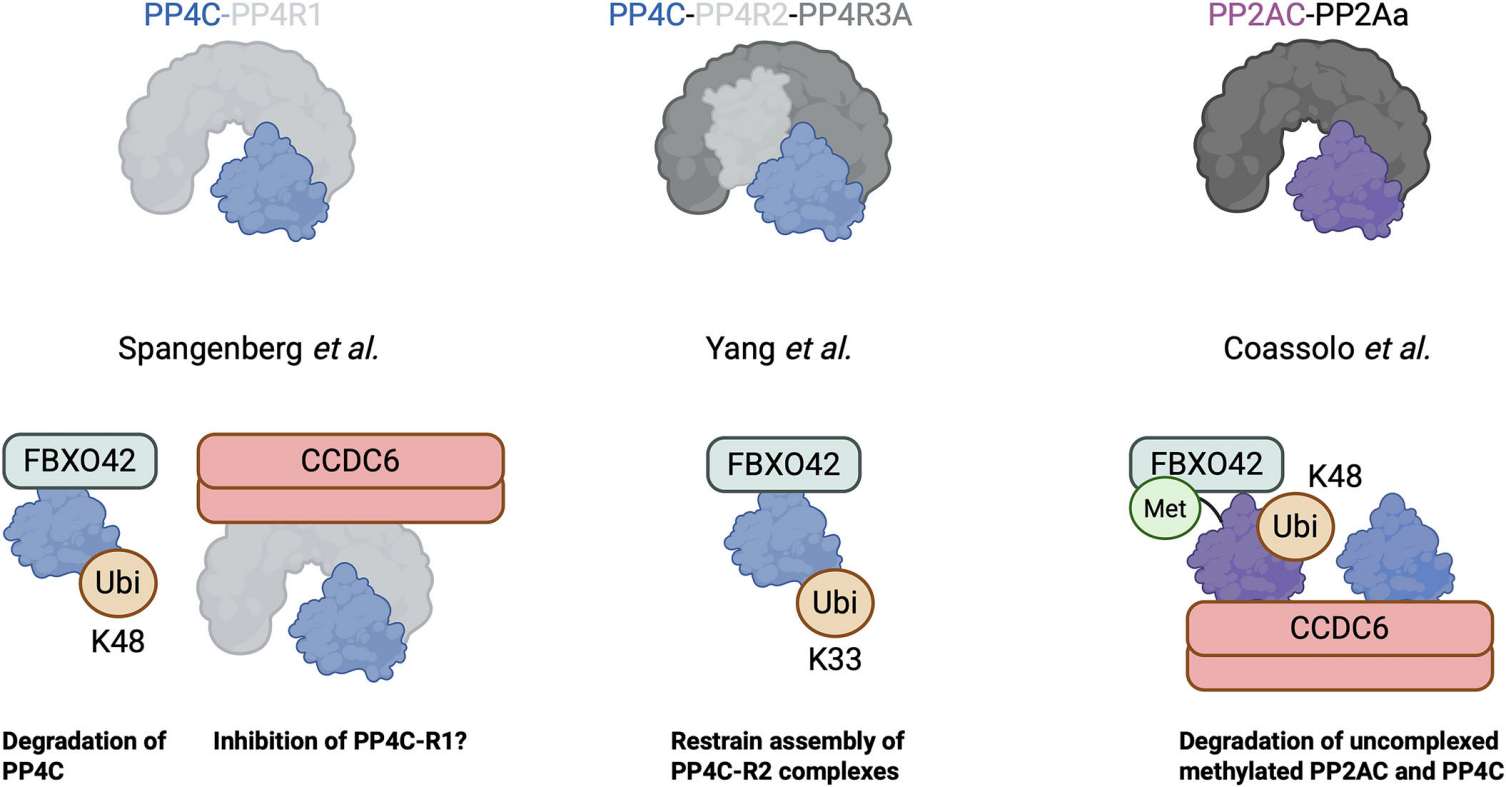
Overview of complexes and proposed mechanisms of FBXO42 and CCDC6. Schematic of PP4 and PP2A complexes discussed and the three models proposed in the respective studies. Figure created in https://BioRender.com.

Although all the new studies show that FBXO42 and CCDC6 function to keep PP2A or PP4 activity in check, the molecular mechanisms proposed are quite distinct (Fig. 1). I will briefly outline the main findings in each of the studies and then summarize common themes emerging from the studies.

Continuing their previous work identifying FBXO42 as a novel mitotic regulator (Hundley et al, 2021), the Toczyski lab searches for substrates of endogenous FBXO42 (Spangenberg et al, 2026). Immunopurification of endogenous FBXO42 from wild-type or *FBXO42*−/− cells combined with mass spectrometry analysis uncovers PP4C, PP4R1, PP4R2, and CCDC6 as specific FBXO42 interactors. Subsequent immunoprecipitation experiments from wild-type, *FBXO42*−/− and *CCDC6*−/− cells show that exogenous PP4C can bind independently to FBXO42 and CCDC6 while the interaction between FBXO42 and CCDC6 depends on PP4C. AlphaFold modeling identifies four similar ExxxNxxxK motifs in the coiled coil region of CCDC6 that mediate binding to the PP4C-PP4R1 complex. To support that FBXO42 and CCDC6 independently regulate PP4C the authors deplete CCDC6 by shRNA from *FBXO42*−/− cells which causes a further reduction in cell growth. To determine how FBXO42 regulates PP4 complexes, they investigate the ubiquitination of the identified PP4 components, identifying PP4C as the main FBXO42 substrate. They show that PP4C is ubiquitinated with K48 chains, a ubiquitin chain type that marks proteins for proteasomal degradation which is consistent with the elevated levels of PP4C in *FBXO42*−/− cells. The ubiquitination and degradation of PP4C by FBXO42 appear independent of CCDC6 as PP4C is still ubiquitinated and degraded in *CCDC6*−/− cells, consistent with their interaction studies. Combinatorial shRNA experiments in a glioblastoma cell line dependent on FBXO42 and CCDC6 show that the reduction in cell growth upon their depletion can be almost fully suppressed by the simultaneous silencing of PP4C. This argues that suppression of PP4C activity is a key function of FBXO42 and CCDC6.

The study from the D’Angiolella lab (Yang et al, 2026) focuses on FBXO42, which emerges as a sensitizer to several DNA-damaging agents in genome-wide CRISPR knockout screens. In line with this, the authors show that *FBXO42*−/− cells have a reduction in the phosphorylation of components controlling the DNA damage checkpoint. By combining affinity purification or proximity-dependent ligation approaches coupled with mass spectrometry, they identify CCDC6, PP4 holoenzyme components (PP4C, PP4R1/2/R3A/R3B) as well as the PP2A catalytic and scaffolding subunits as interactors of FBXO42. Importantly, these interactions are dependent on inhibition of Cullin ubiquitin ligase activity, arguing for transient substrate interactions. Indeed, PP4C and PP4R2 protein levels and complex formation are increased in *FBXO42*−/− cells even though the half-life of PP4C is unaffected by the loss of FBXO42. By taking several experimental approaches to determine the direct substrate of FBXO42 they identify PP4C as a consistent target in several cell lines aligning with the results from the Toczyski lab. However, using in vitro ubiquitination assays with ubiquitin variants, they conclude that short ubiquitin chains with K33 linkages are assembled on PP4C. K33 ubiquitin chains do not target proteins for degradation but rather modulate protein function. The elevated levels of PP4C-PP4R2 complex observed in *FBXO42*−/− cells lead the researchers to investigate whether restraining PP4 activity is a central function of FBXO42 to control cellular sensitivity to irradiation. They deplete PP4 subunits in *FBXO42*−/− cells and monitor cell survival and DNA damage checkpoint markers following irradiation. This shows that the consequence of FBXO42 loss of function can be bypassed by depleting PP4C and PP4R2, from which the authors conclude that a central function of FBXO42 is to restrain assembly of these complexes.

The Hsu and Yauch labs’ entry point to investigating FBXO42 function arose from their observation that several p53 null and mutant cancer cell lines depend on FBXO42 function, pinpointing it as an important therapeutic target (Coassolo et al, 2026). In an elegant approach to identify modulators of FBXO42, they conduct a genome-wide CRISPR knockout screen in an *FBXO42*−/− cell line and find that knockout of the gene encoding the PP2A catalytic subunit (*PPP2CA*) restores growth in this cell line. This promoted further investigations into the link between FBXO42 and *PPP2CA*, and the authors show that FBXO42 can ubiquitinate PP2AC and target the nuclear pool for degradation. Importantly, the ubiquitination is dependent on CCDC6, and a ternary complex of FBXO42-CCDC6-PP2AC forms in cells upon proteasome or Cullin ligase inhibition. To understand the molecular details, they solve the cryo-EM structures of CCDC6-PP2AC and CCDC6-PP2AC-FBXO42 complexes which reveals several unique features of the complex. Firstly, CCDC6 is a dimer and each monomer contains five similar ExxxExxxN motifs (the ExxxN part of this aligns to the ExxxN part of the motifs mentioned above) that each can bind to PP2AC, and in vitro, all motifs appear to be required for efficient PP2AC ubiquitination. Interestingly, PP2AC in complex with its scaffolding subunit (PP2Aa) is unable to bind CCDC6. Secondly, FBXO42 engages the PP2AC-CCDC6 complex without making direct contact to CCDC6 but recognizing unique features of PP2AC, including the methylation group on the terminal leucine. The C-terminal tail of PP2AC engages a conserved pocket on the Kelch domain of FBXO42, with the methylation group making specific contacts to FBXO42. AlphaFold modeling and cellular assays extend these observations to PP4C. In conclusion, these results argue that FBXO42 recognizes methylated PP2AC and PP4C that are not part of holoenzymes.

The studies highlight FBXO42 and CCDC6 as important regulators of PP2A and PP4 and collectively support that in the absence of FBXO42 the catalytic subunits accumulate. Although the work from the Toczyski and D’Angiolella labs focuses on PP4C their data do not rule out regulation of PP2AC. The accumulation of PP2AC and PP4C in *FBXO42-/-* cells is most consistent with K48-mediated ubiquitination but does not exclude that other chain types can form in vitro. All studies support the formation of a ternary FBXO42-CCDC6-PP2AC/4C complex that is likely transient and requires inhibition of Cullin ligases to accumulate. The studies show that elevated levels of the catalytic subunits cause lethality in *FBXO42−/−* cells, but whether elevated PP2AC or PP4C is the main driver of this is likely cell line and context-dependent. Several important future questions are sparked by these studies including whether CCDC6 and FBXO42 can also work independently to inhibit PP2AC and PP4C, whether FBXO42-CCDC6-PP2AC/4C complex assembly is regulated and finally a molecular understanding of why p53-deficient cell lines are particular sensitive to loss of FBXO42 and CCDC6.
